# Genomic surveillance of invasive meningococcal disease in the Czech Republic, 2015-2017

**DOI:** 10.1371/journal.pone.0219477

**Published:** 2019-07-11

**Authors:** Pavla Krizova, Michal Honskus

**Affiliations:** National Reference Laboratory for Meningococcal Infections, Centre for Epidemiology and Microbiology, National Institute of Public Health, Prague, Czech Republic; Defense Threat Reduction Agency, UNITED STATES

## Abstract

**Introduction:**

The study presents the results of the genomic surveillance of invasive meningococcal disease (IMD) in the Czech Republic for the period of 2015–2017.

**Material and methods:**

The study set includes all available IMD isolates recovered in the Czech Republic and referred to the National Reference Laboratory for Meningococcal Infections in 2015–2017, a total of 89 *Neissseria meningitidis* isolates—from 2015 (n = 20), 2016 (n = 27), and from 2017 (n = 42). All isolates were studied by whole genome sequencing (WGS).

**Results:**

Serogroup B (MenB) was the most common, followed by serogroups C, W, and Y. Altogether 17 clonal complexes were identified, the most common of which was hypervirulent complex cc11, followed by complexes cc32, cc41/44, cc269, and cc865. Over the three study years, hypervirulent cc11 (MenC) showed an upward trend. The WGS method showed two clearly differentiated clusters of *N*. *meningitidis* C: P1.5,2:F3-3:ST-11 (cc11). The first cluster is represented by nine isolates, all of which are from 2017. The second cluster consisted of five isolates from 2016 and eight isolates from 2017. Their genetic discordance is illustrated by the changing *nadA* allele and subsequently by the variance in BAST type. Clonal complex cc269 (MenB) also increased over the time frame. WGS identified the presence of MenB vaccine antigen genes in all B and non-B isolates of *N*. *meningitidis*. Altogether 49 different Bexsero antigen sequence types (BAST) were identified and 10 combinations of these have not been previously described in the PubMLST database.

**Conclusions:**

The genomic surveillance of IMD in the Czech Republic provides data needed to update immunisation guidelines for this disease. WGS showed a higher discrimination power and provided more accurate data on molecular characteristics and genetic relationships among invasive *N*. *meningitidis* isolates.

## Introduction

Invasive meningococcal disease (IMD) has one of the highest case fatality rates worldwide despite the recent advances in medicine. The average case fatality rate of this disease is 10% [1], but some hypervirulent clonal complexes (cc) can cause death in up to 25% of cases. The European Centre for Disease Prevention and Control recommends implementing whole genome sequencing (WGS) in the surveillance of infectious diseases, as the most appropriate method to monitor the molecular characteristics of pathogens such as *Neisseria meningitidis* [2]. WGS has already been used in IMD surveillance in some countries [3, 4, 5, 6].

The most effective prevention of IMD is vaccination, and several meningococcal vaccines are currently available. In the Czech Republic, the quadrivalent meningococcal conjugate vaccines (MCV4) and vaccines against *N*. *meningitidis* B (MenB vaccines), a four-component vaccine (4CMenB) and a two-component vaccine (MenB-fHbp), are authorised for use. Given the low incidence of IMD in the Czech Republic [7], the immunisation against meningococcal disease is not included in the national immunisation program, but individual protection is recommended by the Czech Vaccinology Society [8]. Since January 2018, vaccination against IMD is also promoted by the new Czech legislation in individuals with a health indication [9].

Meningococcal vaccines are effective in preventing IMD, but it is necessary to monitor the potential coverage of MenB vaccines against the strains that cause this serious disease. WGS detects, among others, MenB vaccine antigen genes, identifies Bexsero antigen sequence types (BAST, combinations of peptide variants of MenB vaccine antigen genes), and is helpful in the prediction of vaccine coverage of *N*. *meningitidis* isolates by MenB vaccines [10, 11, 12, 13].

The National Reference Laboratory for Meningococcal Infections (NRL) in Prague implemented WGS for *N*. *meningitidis* in 2016, and its use was considered for the nationwide program of IMD surveillance [14]. The feasibility of WGS for this purpose in the Czech Republic was tested on 20 IMD isolates from 2015. In comparison with the conventional sequencing, WGS data provided more accurate information on molecular characteristics of isolates in addition to providing potential coverage estimates with new MenB vaccines [15].

The aim of this study is to present the results of the genomic surveillance of IMD in the Czech Republic for the period 2015–2017, which will improve molecular surveillance achieved by classical sequencing. The reason for investigating IMD isolates from this period by WGS was the increase of MenC which started in the country recently.

## Material and methods

### *Neisseria meningitidis* isolates

All isolates analysed by WGS are from the NRL strain collection which contains more than 1850 *N*. *meningitidis* isolates cultured from IMD cases diagnosed in the Czech Republic during 1971–2018. IMD isolates are referred to the NRL for confirmation and further characterisation in accordance with Czech legislation. *N*. *meningitidis* isolates are stored lyophilised and frozen (-80° C, Cryobank B, ITEST). The *N*. *meningitidis* strain collection electronic database includes clinical, epidemiological and microbiological data on each isolate.

The present genomic study includes all IMD isolates (n = 89) recovered in the Czech Republic and referred to the NRL in 2015–2017, from 2015 (n = 20), 2016 (n = 27), and from 2017 (n = 42). These 89 *N*. *meningitidis* isolates represent 56% of the total of 159 IMD cases reported in this period: from 2015 (n = 48), 2016 (n = 43), and from 2017 (n = 68) and covered 13 out of 14 regions of the Czech Republic and all age groups (S1 Table).

### Identification and characterisation of *Neisseria meningitidis*

The methods used in the present study have been described in detail previously [16]. The isolates from 2016 and 2017 intended for sequencing (n = 65) were plated on chocolate Mueller-Hinton agar and cultured at 37° C and 5% CO_2_ for 18–24 hours. The isolates were assigned to serogroups by conventional serological methods (Pastorex Meningitidis Bio-RAD, antisera *N*. *meningitidis* ITEST, Bio-RAD) and confirmed by RT- PCR. The next step was the isolation of DNA, using the QIAamp DNA Mini Kit, (QIAGEN). WGS was conducted by the European Molecular Biology Laboratory (EMBL), Heidelberg, Germany, using the Illumina MiSeq platform. WGS data was subsequently processed and optimised, using the Velvet *de novo* Assembler software with Velvet-Optimiser [17].

The sixty-five genome contigs were submitted to the *Neisseria* PubMLST database (www.pubmlst.org/neisseria/) under the following IDs: 27059, 27064, 57827, 57828, 57830, 57831, 57833, 37835, 57837–57840, 83803–83817, 83819–83822, 83836–83852, 83866, 83867, 83873, 83878, 83879, 83881–83887, 83890–83894. The previously sequenced isolates from 2015 [15] were also included in this surveillance study (n = 24, IDs: 35105, 35107, 36325, 36329, 36673, 36674, 38267, 38268, 38276, 38278, 38897, 38899, 38901, 38989, 38990, 40373, 40376, 40377, 41191, 41412, 57212, 57213, 57217, 57829). In the PubMLST database, the genome contigs of individual isolates were automatically scanned and the allelic profile of the MLST genes (*abcZ*, *adk*, *aroE*, *fumC*, *gdh*, *pdhC*, *pgm*) determined, assigning sequence type (ST) and clonal complex [18]. Allelic variants were determined in variable regions (VR) contained in the finetyping genes (*porA* and *fetA*). Furthermore, allelic and peptide variants of MenB vaccine antigens (*nhba*, *nadA*, and *fhbp*) were determined [19, 20, 21, 22, 23]. A BAST type is a unique combination of peptide variants of the products of these genes and the two PorA protein variable regions [10].

Genomes were then analysed and compared using the BIGSdb Genome Comparator tool [24] using the core genome cgMLST scheme v1.0 for *N*. *meningitidis* (1605 loci) [25]. Distance matrices based on the number of allelic differences between each pair of isolates were generated automatically and phylogenetic networks constructed and edited using the SplitsTree4 software [26] and the Inkscape tool (www.inkscape.org/en/).

## Results

**Fig 1:**
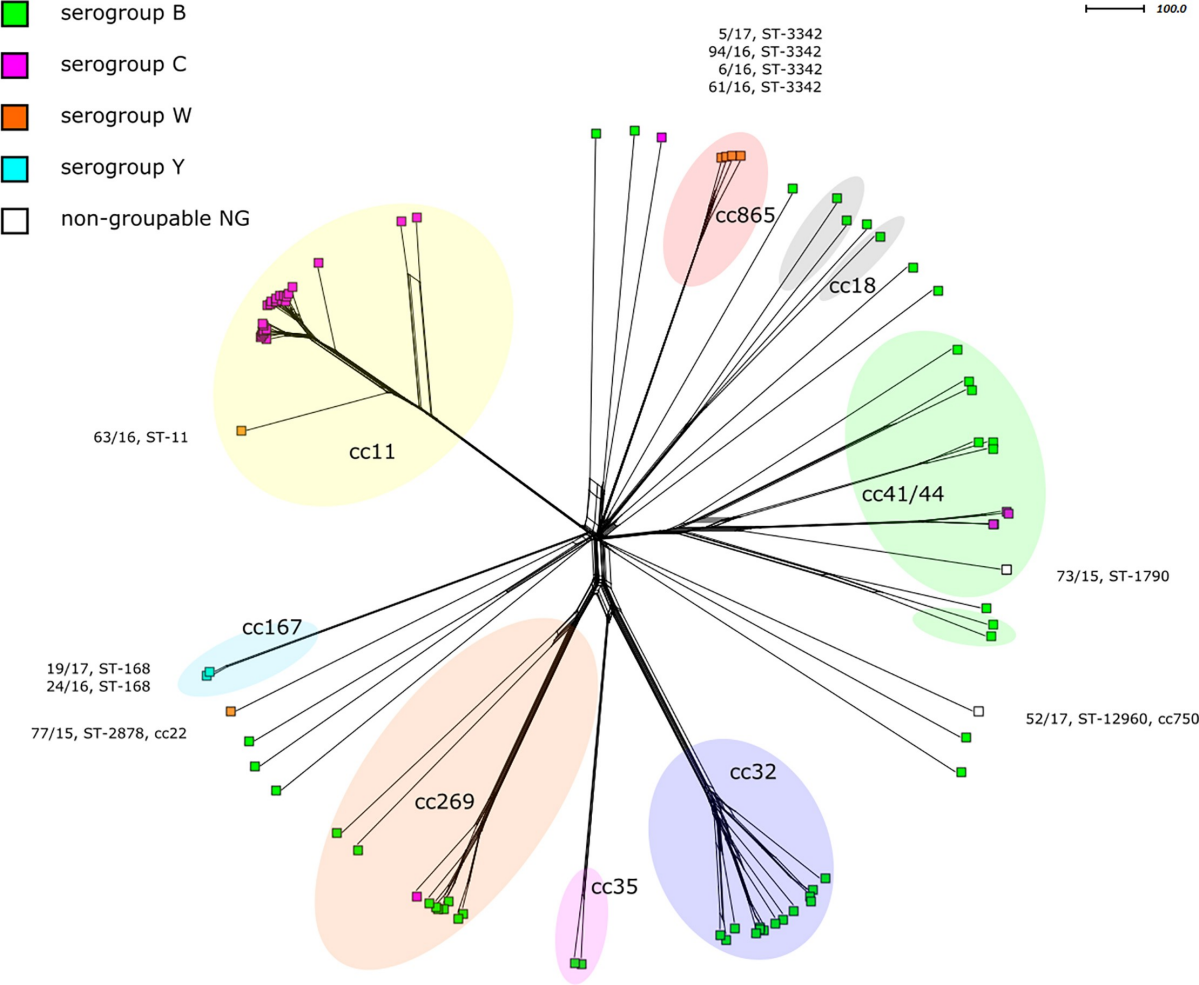
Genetic relationship of *N*. *meningitidis* isolates from invasive meningococcal disease collected in the Czech Republic from 2015 to 2017, (n = 89). A cgMLST Neighbour-net network showing the relatedness among the 89 invasive study isolates. Isolates are coloured according to their serogroup. Only MenW, MenY and MenNG isolates (n = 10) are described by their NRL number, cc and ST. MenB and MenC isolates are shown in details on Fig 2 and Fig 3.

In the study set of 89 *N*. *meningitidis* isolates from IMD cases diagnosed in 2015–2017, most were identified as serogroup B (MenB) (n = 48), followed by serogroup C (MenC) (n = 31), W (MenW) (n = 6), and Y (MenY) (n = 2). Two isolates could not be serogrouped by serological methods—*N*. *meningitidis* non-groupable (MenNG). In both cases, capsular genes were detected by WGS. The occurrence of several mutated and not yet described allelic variants, especially in capsular transport proteins, could explain the inability to include these isolates in specific serogroups. The study of capsular genes and other virulence factors will be the aim of our further research. In total, 17 clonal complexes were identified, with hypervirulent cc11 being the most common, followed by complexes cc32, cc41/44, cc269, and cc865 (Table 1, Fig 1).

**Table 1:** Serogroups and clonal complexes of *N*. *meningitidis* isolates from IMD cases collected in the Czech Republic from 2015 to 2017.

Clonal complex	2015	2016	2017	Total
**MenB**	**14**	**14**	**20**	**48**
**32**	3	5	6	14
**269**	2	0	7	9
**41/44**	2	3	3	8
**18**	1	1	1	3
**35**	2	0	0	2
**162**	1	0	0	1
**60**	1	0	0	1
**213**	1	0	0	1
**334**	0	1	0	1
**174**	0	1	0	1
**1157**	0	0	1	1
**UA**	1	3	2	6
**MenC**	**4**	**8**	**19**	**31**
**11**	2	6	17	25
**41/44**	2	1	1	4
**269**	0	1	0	1
**103**	0	0	1	1
**MenW**	**1**	**4**	**1**	**6**
**865**	0	3	1	4
**11**	0	1	0	1
**22**	1	0	0	1
**MenY**	**0**	**1**	**1**	**2**
**167**	0	1	1	2
**MenNG**	**1**	**0**	**1**	**2**
**41/44**	1	0	0	1
**750**	0	0	1	1
**Total**	**20**	**27**	**42**	**89**

### Serogroup B

Forty-eight MenB isolates were included in the study (Table 1). The most common clonal complex was cc32 (n = 14). The second leading complex was cc269 (n = 9), with seven of these IMD isolates identified in 2017. Six MenB isolates were not assigned to any clonal complex (ccUA).

**Fig 2:**
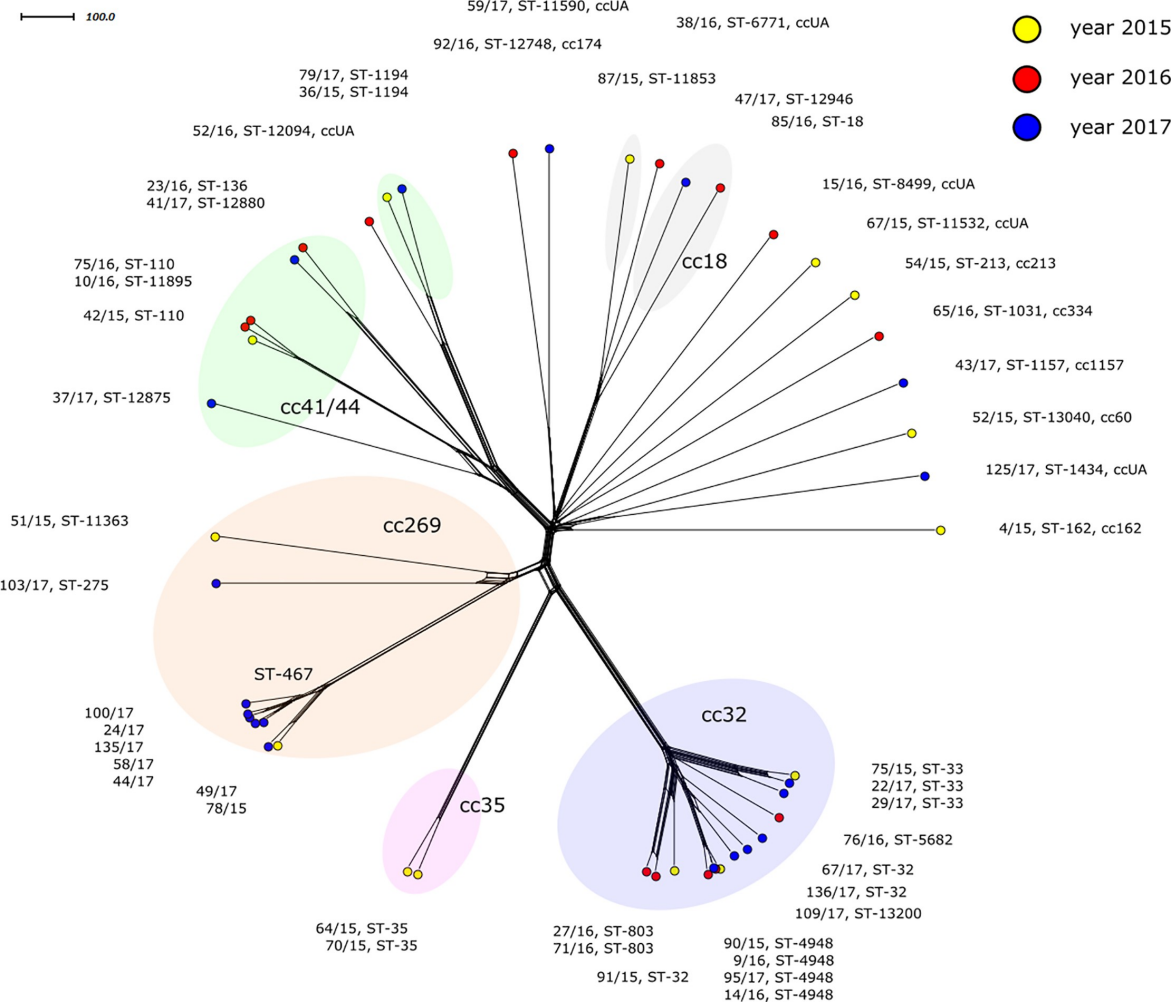
Genetic relationship of *N*. *meningitidis* B isolates from IMD cases collected in the Czech Republic from 2015 to 2017, (n = 48). A cgMLST Neighbour-net network showing the relatedness among the 48 studied invasive MenB isolates. Isolates are coloured according to detection year and labelled by their NRL number, cc and ST.

In the phylogenetic network of MenB isolates, a separate clonal complex, cc32, can be observed (Fig 2). In the table presenting molecular characteristics, the common feature for all cc32 isolates was the presence of peptide variant 1 in two 4CMenB vaccine antigens–NadA and fHbp (Table 2). A third 4CMenB vaccine antigen, NHBA, tended to be peptide variant 3. Isolate 136/17 carried the newly described allelic variant of the *nhba* gene, 1485, which encodes a new peptide variant, 1333.

**Table 2:** Molecular characterization of *N*. *meningitidis* B isolates from IMD cases collected in the Czech Republic from 2015 to 2017.

No. of strain	PubMLST ID	CC	ST	*porA* VR1	*porA* VR2	*fetA* VR	*nhba*	*nhba* peptide	*nadA*	*nadA* variant	*nadA* peptide	*fhbp*	*fhbp* peptide	*fhbp* variant	*fhbp* subfamily	BAST type
**75/15**	38901	**32**	**33**	19	15	F5-1	5	**3**	1	NadA-1	**1**	1	**1**	1	B	**5**
**90/15**	40376	**32**	**4948**	7	16	F3-3	5	**3**	1	NadA-1	**1**	1	**1**	1	B	**4**
**91/15**	40377	**32**	**32**	7	16–20	F3-3	25	**5**	1	NadA-1	**1**	1	**1**	1	B	**79**
**9/16**	83803	**32**	**4948**	7	16	F3-3	5	**3**	1	NadA-1	**1**	1	**1**	1	B	**4**
**14/16**	41412	**32**	**4948**	7	16	F3-3	5	**3**	1	NadA-1	**1**	1	**1**	1	B	**4**
**27/16**	83807	**32**	**803**	7	14	F3-3	25	**5**	1	NadA-1	**1**	1	**1**	1	B	**2991**
**71/16**	83848	**32**	**803**	7	14	F3-3	25	**5**	1	NadA-1	**1**	1	**1**	1	B	**2991**
**76/16**	83850	**32**	**5682**	19	15	F5-1	5	**3**	1	NadA-1	**1**	301	**1**	1	B	**5**
**22/17**	83836	**32**	**33**	19	15	F5-1	5	**3**	1	NadA-1	**1**	1	**1**	1	B	**5**
**29/17**	83838	**32**	**33**	19	15	F5-1	5	**3**	1	NadA-1	**1**	1	**1**	1	B	**5**
**67/17**	83847	**32**	**32**	7–2	30–4	F3-3	5	**3**	1	NadA-1	**1**	1	**1**	1	B	**2994**
**95/17**	83881	**32**	**4948**	7–2	16	F3-3	5	**3**	1	NadA-1	**1**	1	**1**	1	B	**84**
**109/17**	83886	**32**	**13200**	7	16	F3-3	5	**3**	1	NadA-1	**1**	1	**1**	1	B	**4**
**136/17**	83894	**32**	**32**	7	16	F3-3	1485	**1333**	1	NadA-1	**1**	1	**1**	1	B	**3036**
**51/15**	36674	**269**	**11363**	22	14–6	F4-3	0	**0**	0	0	**0**	25	**25**	2	A	**3077**
**78/15**	38990	**269**	**467**	19–1	15–11	F1-7	0	**0**	0	0	**0**	15	**15**	1	B	**3078**
**24/17**	83837	**269**	**467**	19–1	15–11	F1-7	14	**21**	0	0	**0**	15	**15**	1	B	**222**
**44/17**	83842	**269**	**467**	19–1	15–11	F1-7	14	**21**	0	0	**0**	15	**15**	1	B	**222**
**49/17**	83844	**269**	**467**	19–1	15–11	F1-7	906	**870**	0	0	**0**	15	**15**	1	B	**2983**
**58/17**	83845	**269**	**467**	19–1	15–11	F1-7	14	**21**	0	0	**0**	15	**15**	1	B	**222**
**100/17**	83883	**269**	**467**	19–1	15–11	F1-7	14	**21**	0	0	**0**	15	**15**	1	B	**222**
**103/17**	83885	**269**	**275**	22	9	F5-12	18	**17**	0	0	**0**	19	**19**	2	A	**267**
**135/17**	83893	**269**	**467**	19–1	15–11	F1-7	14	**21**	0	0	**0**	15	**15**	1	B	**222**
**36/15**	36325	**41/44**	**1194**	18–1	3	F3-9	1	**2**	0	0	**0**	4	**4**	1	B	**239**
**42/15**	36329	**41/44**	**110**	19	15	F1-7	1	**2**	0	0	**0**	19	**19**	2	A	**644**
**10/16**	83804	**41/44**	**11895**	19–2	15–10	F1-7	1	**2**	0	0	**0**	19	**19**	2	A	**2976**
**23/16**	83806	**41/44**	**136**	17	16–3	F5-5	35	**10**	0	0	**0**	24	**24**	2	A	**253**
**75/16**	83849	**41/44**	**110**	19	15	F1-7	1	**2**	0	0	**0**	19	**19**	2	A	**644**
**37/17**	83839	**41/44**	**12875**	21–2	28	F3-6	1484	**1332**	0	0	**0**	1444	**207**	ND	A/B	**3035**
**41/17**	83840	**41/44**	**12880**	17	16–3	F1-47	35	**10**	0	0	**0**	1445	**1114**	2	A	**2981**
**79/17**	83879	**41/44**	**1194**	18–1	3	F1-5	1	**2**	0	0	**0**	4	**4**	1	B	**239**
**87/15**	40373	**18**	**11853**	22	14	F5-5	9	**6**	0	0	**0**	36	**37**	1	B	**247**
**85/16**	83851	**18**	**18**	22	14–6	F5-77	923	**883**	0	0	**0**	36	**37**	1	B	**2980**
**47/17**	83843	**18**	**12946**	12–1	16	F3-9	9	**6**	0	0	**0**	36	**37**	1	B	**2982**
**64/15**	38276	**35**	**35**	22–1	14	F4-1	19	**21**	0	0	**0**	16	**16**	2	A	**257**
**70/15**	38897	**35**	**35**	22–1	14	F5-18	19	**21**	0	0	**0**	16	**16**	2	A	**257**
**4/15**	35107	**162**	**162**	7–2	4	F5-9	11	**20**	0	0	**0**	21	**21**	2	A	**246**
**52/15**	38267	**60**	**13040**	5	2	F5-1	15	**24**	0	0	**0**	13	**13**	1	B	**237**
**54/15**	38268	**213**	**213**	22	14	F5-5	33	**18**	40	NadA-4/5	**0**	44	**59**	3	A	**304**
**65/16**	83810	**334**	**1031**	7–4	14–6	F5-2	9	**6**	0	0	**0**	13	**13**	1	B	**2978**
**92/16**	83852	**174**	**12748**	21	16	F4-1	280	**81**	0	0	**0**	349	**296**	2	A	**472**
**43/17**	83841	**1157**	**1157**	21–7	16	F5-36	66	**114**	20	NadA-2/3	**0**	68	**13**	1	B	**271**
**67/15**	38278	**UA**	**11532**	5–3	2–16	F5-5	294	**63**	0	0	**0**	1170	**931**	1	B	**2555**
**15/16**	83805	**UA**	**8499**	5–3	2–16	F3-9	926	**180**	0	0	**0**	36	**37**	1	B	**2977**
**38/16**	83808	**UA**	**6771**	22	14	F5-8	257	**89**	84	NadA-4/5	**92**	36	**37**	1	B	**2992**
**52/16**	83809	**UA**	**12094**	7–2	4	F5-2	1	**2**	0	0	**0**	14	**14**	1	B	**223**
**59/17**	83846	**UA**	**11590**	17	16–3	F1-216	1439	**1298**	0	0	**0**	102	**102**	2	A	**2984**
**125/17**	83891	**UA**	**1434**	5–1	2–2	F5-5	352	**306**	0	0	**0**	16	**16**	2	A	**815**

CC = clonal complex; ST = sequence type; ccUA = clonal complex unassigned; *porA* VR1, VR2 = *porA* variable region 1 and 2; *fetA* VR = *fetA* variable region; 0 = isolate lacks a functional allele; yellow highlight = newly described sequence type, or the BAST type; green highlight = potential cross reactive MenB vaccine antigens [32]

Seven of the cc269 isolates, all assigned to ST-467, showed high relatedness to each other (Fig 2). Isolates 51/15, ST-11363 and 103/17, ST-275 were genetically distant from the ST-467 cluster. The ST-467 isolates had an identical finetyping profile (P1.19–1,15–11:F1-7) and fHbp peptide variant 15 (Table 2). Apart from isolate 49/17 which carries NHBA peptide variant 870 and isolate 78/15 where the *nhba* allele was not detected, the ST-467 isolates have the same NHBA peptide variant 21.

Eight of the Men B isolates were assigned to clonal complex cc41/44. This complex is genetically rather heterogeneous in the phylogenetic network (Fig 2). A feature common to all cc41/44 isolates was the absence of the *nadA* gene (Table 2). Isolate 37/17 possessed a new allelic and peptide *nhba* variant.

Clearly separated but genetically more distant were cc18 isolates (87/15, ST-11853, 85/16, ST-18, and 47/17, ST-12946), along with isolate 38/16, ST-6771 (ccUA) (Fig 2). Molecular characteristics of cc18 isolates were identical in *fhbp* variant 36 encoding peptide variant 37 and in the absence of the *nadA* gene (Table 2).

Distant relatedness can also be seen in the phylogenetic network between isolates 92/16, ST-12748 (cc174) and 59/17, ST-11590 (ccUA) (Fig 2). Other MenB isolates did not show any relatedness to each other or to any cluster of a known clonal complex. The only element of the antigen genes in Table 2 that the isolates (cc162, cc60, cc213, cc334, cc174, and cc1167) have in common was the absence of the *nadA* protein product. The reason in most cases was the complete absence of the *nadA* gene; two isolates (54/15 and 43/17) carried an allelic variant which does not produce a functional protein due to a shifted reading frame.

In all MenB study isolates, WGS detected MenB vaccine antigen genes and BAST types which were highly diverse (Table 2). Altogether 34 different BAST types were identified, and eight combinations of these had not been previously described in the PubMLST database. WGS detected two new *nhba* alleles and peptide variants and a new allele of the *aroE* gene, which made it possible to describe a new ST, 13040, in isolate 52/15.

### Serogroup C

The study group included 31 MenC isolates (Table 1). Most of these isolates were assigned to clonal complex cc11 (n = 25). Four isolates were assigned to cc41/44 and only two isolates belonged to other clonal complexes: 39/16 (cc269) and 57/17 (cc103).

**Fig 3:**
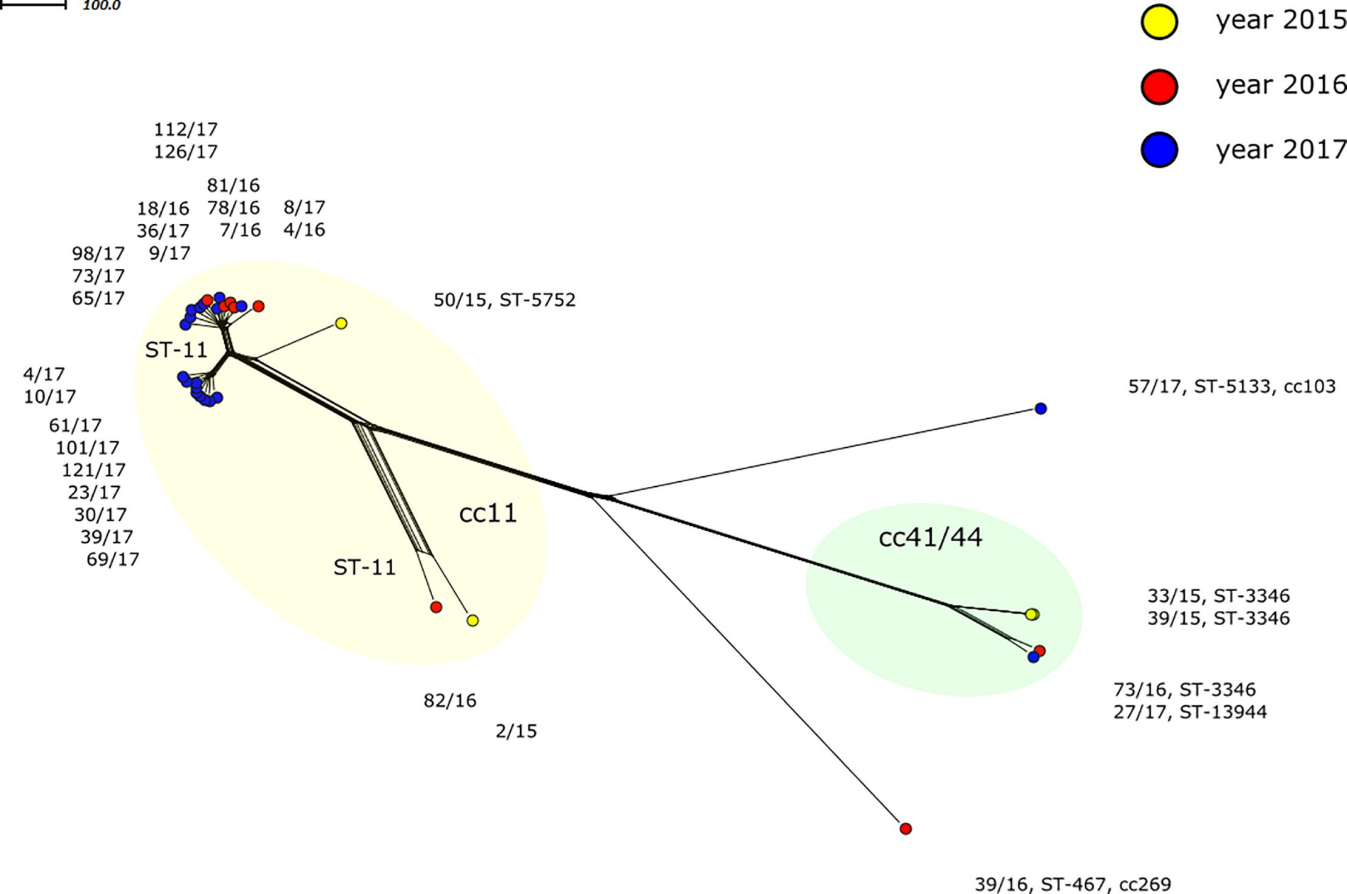
Genetic relationship of *N*. *meningitidis* C isolates from IMD cases collected in the Czech Republic from 2015 to 2017, (n = 31). A cgMLST Neighbour-net network showing the relatedness among the 31 invasive MenC isolates studied. Isolates are coloured according to detection year and labelled by their NRL number, cc and ST.

Almost all MenC cc11 isolates, except 50/15, ST-5752, were assigned to ST-11 (Fig 3). Most C: P1.5,2:F3-3:ST-11 (cc11) isolates formed two genetically close but clearly separated clusters. Cluster 1 grouped nine isolates, all of which were from 2017. Cluster 2 included five isolates from 2016 and eight isolates from 2017. The above-mentioned ST-5752 isolate 50/15 from 2015 showed partial relatedness to the two clusters. It is evident from the table of molecular characteristics (Table 3) that isolates of two largest and highly related clusters shared nearly all characteristics. Their genetic discordance reflected by their distribution into two separated clusters was illustrated by the *nadA* allele. Cluster 1 grouping exclusively isolates from 2017 is characterised by allele 117 producing peptide 121. For this reason, all cluster 1 isolates were assigned to BAST 8. Cluster 2 isolates were carrying NadA peptide variant 3 and thus assigned to BAST 3. The only exception is isolate 98/17, in which no *porA* allele was detected and which was assigned to BAST 830. Isolate 50/15, ST-5752 differed in the *abcZ* gene where a single-nucleotide change resulted in replacement of allele 2 by allele 370. Another difference was *nadA* allelic variant 140 encoding peptide 127. Isolates 2/15 and 82/16, which formed a clearly genetically distant lineage, were distinguished from all other ST-11 (cc11) isolates by *nhba* allele 3 (peptide 20) and the absence of the *nadA* allele.

**Table 3:** Molecular characterization of *N*. *meningitidis* C isolates from IMD cases collected in the Czech Republic from 2015 to 2017.

No. of strain	PubMLST ID	CC	ST	*porA* VR1	*porA* VR2	*fetA* VR	*nhba*	*nhba* peptide	*nadA*	*nad*A variant	*nad*A peptide	*fhbp*	*fhbp* peptide	*fhbp* variant	*fhbp* subfamily	BAST type
**2/15**	35105	**11**	**11**	5	2	F3-6	3	**20**	0	0	**0**	1511	**1156**	1	B	**2985**
**50/15**	36673	**11**	**5752**	5	2	F3-3	17	**29**	140	NadA-2/3	**127**	22	**22**	2	A	**38**
**4/16**	57827	**11**	**11**	5	2	F3-3	17	**29**	3	NadA-2/3	**3**	22	**22**	2	A	**3**
**7/16**	57830	**11**	**11**	5	2	F3-3	17	**29**	3	NadA-2/3	**3**	22	**22**	2	A	**3**
**18/16**	57833	**11**	**11**	5	2	F3-3	17	**29**	3	NadA-2/3	**3**	22	**22**	2	A	**3**
**78/16**	57838	**11**	**11**	5	2	F3-3	17	**29**	3	NadA-2/3	**3**	22	**22**	2	A	**3**
**81/16**	57839	**11**	**11**	5	2	F3-3	17	**29**	3	NadA-2/3	**3**	22	**22**	2	A	**3**
**82/16**	57840	**11**	**11**	5	2	F1-7	3	**20**	0	0	**0**	1448	**1116**	1	B	**2979**
**4/17**	57828	**11**	**11**	5	2	F3-3	17	**29**	117	NadA-2/3	**121**	22	**22**	2	A	**8**
**8/17**	57831	**11**	**11**	5	2	F3-3	17	**29**	3	NadA-2/3	**3**	22	**22**	2	A	**3**
**9/17**	83811	**11**	**11**	5	2	F3-3	17	**29**	3	NadA-2/3	**3**	22	**22**	2	A	**3**
**10/17**	83812	**11**	**11**	5	2	F3-3	17	**29**	117	NadA-2/3	**121**	22	**22**	2	A	**8**
**23/17**	83813	**11**	**11**	5	2	F3-3	17	**29**	117	NadA-2/3	**121**	22	**22**	2	A	**8**
**30/17**	83815	**11**	**11**	5	2	F3-3	17	**29**	117	NadA-2/3	**121**	22	**22**	2	A	**8**
**36/17**	83816	**11**	**11**	5	2	F3-3	17	**29**	3	NadA-2/3	**3**	22	**22**	2	A	**3**
**39/17**	83817	**11**	**11**	5	2	F3-3	17	**29**	117	NadA-2/3	**121**	22	**22**	2	A	**8**
**61/17**	83820	**11**	**11**	5	2	F3-3	17	**29**	117	NadA-2/3	**121**	22	**22**	2	A	**8**
**65/17**	83821	**11**	**11**	5	2	F3-3	17	**29**	3	NadA-2/3	**3**	22	**22**	2	A	**3**
**69/17**	83822	**11**	**11**	5	2	F3-3	17	**29**	117	NadA-2/3	**121**	22	**22**	2	A	**8**
**73/17**	83878	**11**	**11**	5	2	F3-3	17	**29**	3	NadA-2/3	**3**	22	**22**	2	A	**3**
**98/17**	83882	**11**	**11**	0	0	F3-3	17	**29**	3	NadA-2/3	**3**	22	**22**	2	A	**830**
**101/17**	83884	**11**	**11**	5	2	F3-3	17	**29**	117	NadA-2/3	**121**	22	**22**	2	A	**8**
**112/17**	83887	**11**	**11**	5	2	F3-3	17	**29**	3	NadA-2/3	**3**	22	**22**	2	A	**3**
**121/17**	83890	**11**	**11**	5	2	F3-3	17	**29**	117	NadA-2/3	**121**	22	**22**	2	A	**8**
**126/17**	83892	**11**	**11**	5	2	F3-3	17	**29**	3	NadA-2/3	**3**	22	**22**	2	A	**3**
**33/15**	27059	**41/44**	**3346**	17	16–4	F3-4	300	**188**	0	0	**0**	14	**14**	1	B	**1071**
**39/15**	27064	**41/44**	**3346**	17	16–4	F3-4	300	**188**	0	0	**0**	14	**14**	1	B	**1071**
**73/16**	57837	**41/44**	**3346**	17	16–4	F3-9	300	**188**	0	0	**0**	14	**14**	1	B	**1071**
**27/17**	83814	**41/44**	**13944**	17	16–4	F3-9	300	**188**	0	0	**0**	14	**14**	1	B	**1071**
**39/16**	57835	**269**	**467**	19–1	15–11	F1-7	14	**21**	0	0	**0**	15	**15**	1	B	**222**
**57/17**	83819	**103**	**5133**	0	0	F3-9	15	**24**	0	0	**0**	19	**19**	2	A	**1010**

CC = clonal complex; ST = sequence type; ccUA = clonal complex unassigned; *porA* VR1, VR2 = *porA* variable region 1 and 2; *fetA* VR = *fetA* variable region; 0 = isolate lacks a functional allele; yellow highlight = newly described sequence type; green highlight = potential cross reactive MenB vaccine antigens [32]

Four serogroup C isolates were assigned to clonal complex cc41/44, which is genetically distant from cc11 (Fig 3). Most of these isolates were assigned to ST-3346 (n = 3). Apart from the *fetA* gene whose VR harbours two different peptide variants (F3-4 and F3-9), isolates cc41/44 shared all characteristics and were assigned to the same BAST (Table 3). Isolate 27/17 underwent a single-nucleotide change resulting in the replacement of the *adk* gene, where the initial allele 6 was replaced by a newly described allele, 660. This resulted in a new sequence type, ST-13944.

In the phylogenetic network, isolates 39/16, ST-467 (cc269) and 57/17, ST-5133 (cc103) can also be seen (Fig 3). These isolates were genetically very distant from clonal complexes cc11 and cc41/44 and did not show any relatedness to each other (Table 3).

In all MenC study isolates, WGS detected MenB vaccine antigen genes, and the isolates were assigned to BAST types which were highly homogeneous as compared with the MenB isolates. Altogether nine previously described BAST types were identified.

### Serogroups W and Y and *N*. *meningitidis* NG

The study set included MenW isolates (n = 6), four of which were assigned to clonal complex cc865 (Table 1), uncommon for serogroup W. All cc865 isolates were assigned to ST-3342, so far reported to the PubMLST database exclusively from the Czech Republic [16]. A single MenW cc11 isolate (63/16) recovered from an imported case of IMD in 2016 was assigned to hypervirulent UK subclone W: P1.5,2:F1-1:ST-11 (cc11). The study set also included MenY isolates (n = 2), both assigned to cc167, and MenNG isolates (n = 2), one of them assigned to cc41/44 and the other to cc750.

In all these isolates (MenW, MenY, and MenNG) WGS detected MenB vaccine antigen genes, and they were assigned to BAST (Table 4). Altogether six different BAST types were identified, and two combinations of these had not been previously described in the PubMLST database.

**Table 4:** Molecular characterization of *N*. *meningitidis* W, Y and NG isolates from IMD cases collected in the Czech Republic from 2015 to 2017.

No. of strain	Serogroup	PubMLST ID	CC	ST	*porA* VR1	*porA* VR2	*fetA* VR	*nhba*	*nhba* peptide	*nadA*	*nad*A variant	*nad*A peptide	*fhbp*	*fhbp* peptide	*fhbp* variant	*fhbp* subfamily	BAST type
**63/16**	**W**	57213	**11**	**11**	5	2	F1-1	17	**29**	5	NadA-2/3	**6**	22	**22**	2	A	**2**
**77/15**	**W**	38989	**22**	**2878**	18–1	3	F4-1	3	**20**	0	0	**0**	16	**16**	2	A	**349**
**6/16**	**W**	41191	**865**	**3342**	5–2	10–1	F5-8	257	**89**	109	NadA-4/5	**21**	380	**321**	1	B	**1320**
**61/16**	**W**	57212	**865**	**3342**	5–2	10–1	F5-8	257	**89**	109	NadA-4/5	**21**	380	**321**	1	B	**1320**
**94/16**	**W**	57217	**865**	**3342**	5–2	10–1	F5-8	257	**89**	109	NadA-4/5	**21**	380	**321**	1	B	**1320**
**5/17**	**W**	57829	**865**	**3342**	5–2	10–1	F5-8	1438	**89**	109	NadA-4/5	**21**	380	**321**	1	B	**1320**
**24/16**	**Y**	83867	**167**	**168**	5–1	10–4	F4-1	509	**9**	0	0	**0**	23	**23**	2	A	**384**
**19/17**	**Y**	83866	**167**	**168**	5–1	10–4	0	509	**9**	0	0	**0**	23	**23**	2	A	**384**
**73/15**	**NG**	38899	**41/44**	**1790**	7	30	F3-5	1	**2**	0	0	**0**	22	**22**	2	A	**2554**
**52/17**	**NG**	83873	**750**	**12960**	7–2	1–5	F3-9	234	**129**	0	0	**0**	1446	**1007**	2	A	**2993**

CC = clonal complex; ST = sequence type; NG = *N*. *meningitidis* non-groupable; ccUA = clonal complex unassigned; *porA* VR1, VR2 = *porA* variable region 1 and 2; *fetA* VR = *fetA* variable region; 0 = isolate lacks a functional allele; yellow highlight = newly described gene allele or the BAST type; green highlight = potential cross reactive MenB vaccine antigens [32]

## Discussion

The high resolution power of WGS provides new possibilities for the analysis of *N*. *meningitidis* for public health purposes. Recently, *N*. *meningitidis* W cc11 has become the main cause of IMD in several European countries. Most cases of IMD in the UK, the Netherlands, Sweden, and France are caused by strains from the same lineages of hypervirulent *N*. *meningitidis* W cc11 [3, 4, 5, 6, 27]. Our WGS study shows that the Czech isolates of *N*. *meningitidis* W do not belong to these hypervirulent cc11 lineages, but belong to clonal complex cc865, are genetically highly homogeneous and present the same sequence type ST-3342 [16]. Based on the data available in the PubMLST database, sequence type ST-3342 has only been reported in the Czech Republic.

One of the aims of our study was to identify if the recent increase of MenC causing IMD is due to homologous clonal complex cc11. Historically, MenB was the predominant cause of IMD in the Czech Republic. However, this changed in the mid-1990s, when clonal complex cc11 MenC emerged: MenC prevailed in the period from 1994 to 1998 (S1 Fig). MenC cc11 isolates caused the increase of incidence of IMD with the peak of 2.2/100 000 population in 1995. After this, the incidence gradually decreased reaching the minimum 0.4/100 000 population in 2014 and 2016 (S2 Fig). The reason for investigation of IMD isolates from the period 2015–2017 by WGS was the increase of MenC which started recently (S1 Fig). IMD isolates are routinely characterized in the NRL by classical sequencing methods and the main clonal complex causing recent increase of MenC was cc11. Strain characterisation based on classical sequencing methods do not afford the necessary resolution to distinguish among the highly clonal sub-lineages of cc11 meningococci [28]. For example, in the UK, a new sub-lineage of MenW isolates (cc11) caused the increase of IMD in 2013 [5]. The cocirculation of different sub-lineages of MenW was published recently from Italy, where the Hajj and the South American sub-lineages of cc11 were gradually replaced by cc22 [29].

The Prague NRL used WGS in 2017 to study a set of 31 Czech isolates of *N*. *meningitidis* W from 1984–2017, and the results have already been published [16]. The most interesting finding of that study was the fact that eight of the 31 *N*. *meningitidis* W isolates were assigned to clonal complex cc865, which is, based on PubMLST data, uncommon among serogroup W isolates. All Czech cc865 isolates are genetically highly homogeneous, were recovered between 2010 and 2017, and are assigned to a single sequence type, ST-3342, which has so far been reported exclusively from the Czech Republic. WGS data on the Czech serogroup W meningococcal isolates confirm the presence of MenB vaccine antigen genes and thus do not disprove the hypothesis that this vaccine has potential for protection against *N*. *meningitidis* W.

The limitation of this study is that 89 isolates from IMD present 56% of 159 cases recorded in the surveillance program in the Czech Republic in 2015–2017. In that period, 25.8% of IMD cases were confirmed by non-culture PCR assay only (isolates from these cases were not available) and laboratory confirmation of IMD was reported to the surveillance system in 18.2% of cases, but the *N*. *meningitidis* isolates were not referred to the NRL.

The molecular characteristics and phylogenetic network show that serogroup B is a heterogeneous population where only three larger groups of isolates can be noticed and are assigned to the following clonal complexes: cc32, cc269, and cc41/44. Even within these groups, the relatedness between isolates varies. A considerable proportion of isolates (17 out of 48) are assigned to clonal complexes represented by few isolates or even by a single isolate as is the case with six clonal complexes. Six isolates were ccUA. During the three-year study period, cc269 (MenB) showed an upward trend. Compared to serogroup B, MenC isolates were clearly less heterogeneous. Most MenC isolates were assigned to clonal complex cc11 and isolates assigned to other clonal complexes were found only sporadically in the Czech Republic. Our study also indicates that MenC isolates belonging to hypervirulent clonal complex cc11 showed an upward trend. Almost all these isolates (21 of 25) exhibit the same molecular characteristics: P1.5,2:F3-3:ST-11. Interestingly, these 21 highly related isolates form two separate clusters in the Czech Republic, which is observable both from their position on the phylogenetic network and from the differences of these isolates in some molecular characteristics. Their genetic discordance is illustrated by the *nadA* allele. Smaller cluster 1 group isolates from 2017 only (n = 9) were characterised by *nadA* allele 117 producing peptide 121 (BAST 8). The larger cluster 2, which contains isolates from 2016 (n = 5) and 2017 (n = 8), was specific to the *nadA* allele 3 and these isolates were assigned to BAST 3. The supplementary table (S1 Table) shows that there is the link with the region where the isolates were detected. Six of nine cluster 1 isolates came from the CZ031 region (South Bohemian region; south). Cluster 2 (n = 13) contained 10 isolates from the neighbouring CZ032 region (Pilsen region; southwest). Thus, two clusters of P1.5,2:F3-3:ST-11 (cc11) isolates represent two regionally specific populations of *N*. *meningitidis* C.

The especially virulent MenC cc11 clones of the 1990s, electrophoretic type (ET) ET-15, were distinguished from other MenC cc11 by the presence of a single point mutation in the fumarase C gene (*fumC*). The point mutation at position 640 is a clone-specific characteristic which permits the distinction of ET-15 (640A) from other ET-37 (640G) complex strains [30]. Our results of WGS analysis showed that the increase of MenC IMD in 2016 and 2017 was caused by two genetically different clusters of cc11, distinguished temporally and geographically, which are different, for example, in the *nadA* allele and consequently their BAST type. All these isolates presented a single point mutation 640G in the *fumC* gene and therefore do not belong to especially virulent ET-15 clones.

The bactericidal activity of the new MenB vaccines on *N*. *meningitidis* isolates can be tested by MATS and MEASURE functional assays [31, 32]. A recent extensive international study showed an alternative method gMATS, which offers comparable coverage estimates to the time consuming functional assays [33]. The genomic surveillance of antigenic variants of the 4CMenB vaccine among IMD isolates from the UK from 2010–2016 showed that before this vaccine was integrated in the UK immunisation program for small infants, 3073 study isolates were assigned to 803 BAST types. WGS data point to cross reactivity of the 4CMenB vaccine antigens and its potential for protection also against non-B meningococci [13]. In our study, WGS data showed the presence of MenB vaccine antigen genes in all study B and non-B isolates of *N*. *meningitidis*, which suggests that the vaccine has potential for protection also against non-B meningococci in the Czech Republic. In the study set of 89 invasive *N*. *meningitidis* isolates from 2015–2017 we observed more than 50% potential coverage by 4CMenB vaccine based on a study with a new gMATS method [33]. In MenB isolates (n = 48), 37 were covered (1 by three antigenic peptides, 21 by two antigenic peptides, 15 by one antigenic peptide). PorA VR2 peptide variant 4 was found in two out of 48 MenB isolates only. In MenC isolates, the potential coverage by a single antigenic peptide showed six isolates and two peptides were detected in one isolate (i.e. 7 out of 31 MenC isolates). In a group of MenW, MenY, and MenNG isolates (n = 10), potential coverage by 4CMenB vaccine was observed only in two isolates (by a single antigenic peptide). Continuing the monitoring of MenB vaccine antigen genes in Czech *N*. *meningitidis* isolates is needed for a qualified prediction of the efficiency of MenB vaccines in the Czech Republic.

## Supporting information

S1 Fig Serogroup frequency in invasive meningococcal disease in the Czech Republic, 1993–2017, surveillance data. MenB, MenC, MenY. MenW.
(XLS)

S2 Fig Invasive meningococcal disease incidence in the Czech Republic, 1993–2017, surveillance data. Incidence per 100 000.
(XLS)

S1 Table Epidemiological data of 89 studied *N. meningitidis* invasive isolates from the Czech Republic collected in 2015, 2016 and 2017. Epidemiological data: year of isolation, age group, region. Region is indicated by Nomenclature of Units for Territorial Statistics.
(XLSX)
